# Supplementary material for: Liposomal Bilayer as a Carrier of Rosa canina L. Seed Oil: Physicochemical Characterization, Stability, and Biological Potential
Source: Molecules. 2022 Dec 29;28(1):276. doi: 10.3390/molecules28010276 (PMC9821806; doi:10.3390/molecules28010276)
Supplement: Supplementary file 1 [file molecules-28-00276-s001.zip › molecules-2067277-supplementary.pdf]

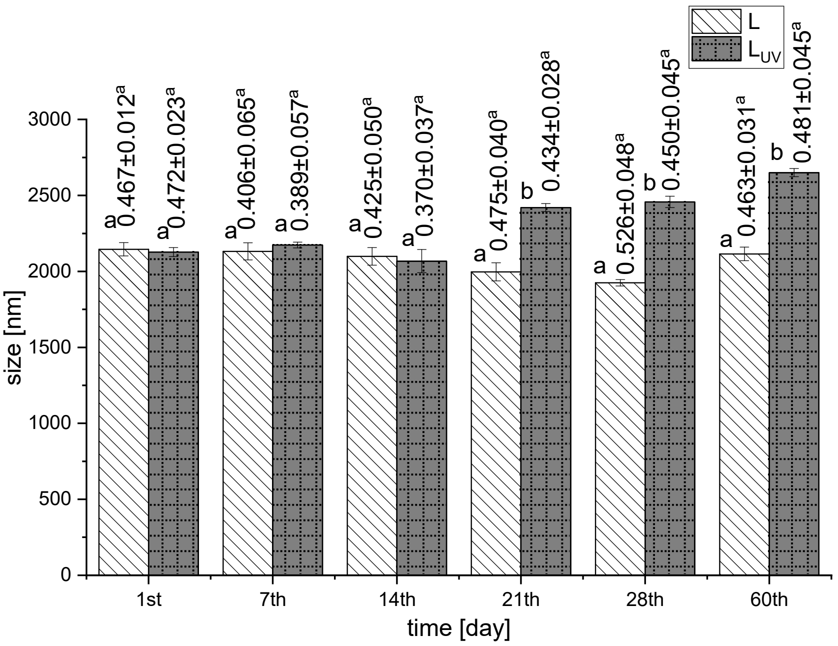


(a)

| conductivity [mS/cm] | | |
|---|---|---|
| | L | $L_{UV}$ |
| 1st | 0.020±0.002a | 0.021±0.002a |
| 7th | 0.021±0.002a | 0.022±0.003a |
| 14th | 0.016±0.002b | 0.016±0.001b |
| 21st | 0.013±0.002b | 0.014±0.002b |
| 28th | 0.012±0.001b | 0.016±0.001b |
| 60th | 0.015±0.002b | 0.015±0.003b |

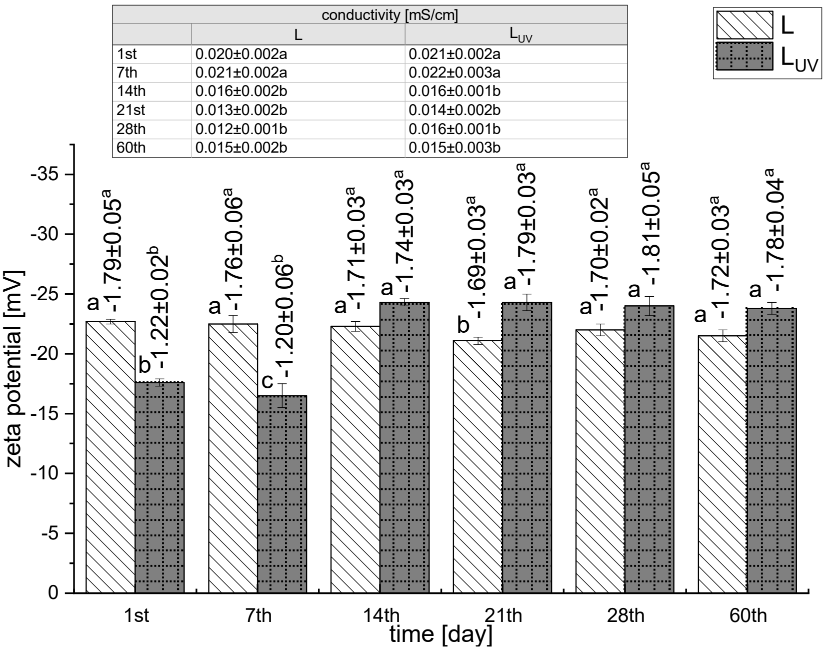


(b)

**Figure S1.** Liposomes size - bars and polydispersity index - numbers above bars (**a**) and zeta potential - bars, mobility (μmcm/Vs) - numbers above bars, and conductivity - table (**b**) of non-treated and UV-irradiated plain liposomes during 60 days storage at 4°C; values with different letters (a-d) in each row showed statistically significant differences ($p<0.05$; n=3; analysis of variance, Duncan's *post-hoc* test); L, liposomes.

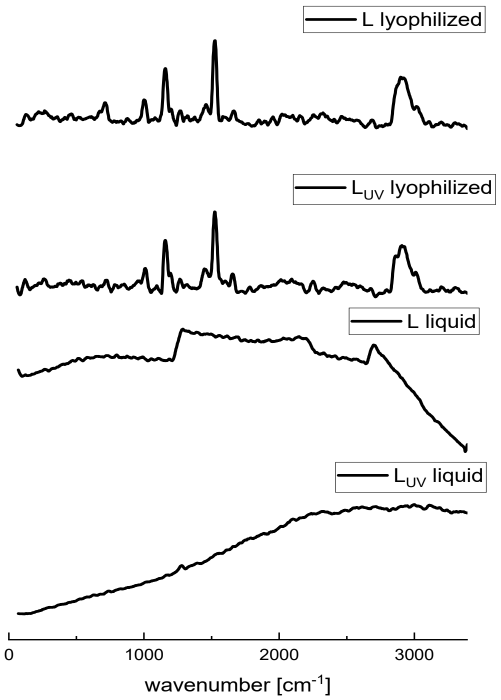


**Figure S2.** Raman spectra of non-treated and UV-irradiated plain liposomes (liquid and lyophilized) in the spectral range from 70 to 3400 cm$^{-1}$.
